# Bis(dicyanamido-κ*N*)tetra­kis­(pyridine-κ*N*)nickel(II)

**DOI:** 10.1107/S1600536812019691

**Published:** 2012-05-12

**Authors:** Susanne Wöhlert, Mario Wriedt, Inke Jess, Christian Näther

**Affiliations:** a Institut für Anorganische Chemie, Christian-Albrechts-Universität Kiel, Max-Eyth-Strasse 2, 24118 Kiel, Germany; b Departement of Chemistry, Texas A&M University, College Station, Texas 77843, USA

## Abstract

In the crystal structure of the title compound, [Ni(C_2_N_3_)_2_(C_5_H_5_N)_4_], the Ni^II^ cations are coordinated by four pyridine ligands and two dicyanamide anions into discrete complexes. The shortest Ni⋯Ni separation is 8.1068 (10) Å. The structure is pseudo-centrosymmetric and can also be refined in the space group *C*2/*c* in which both anionic ligands are strongly disordered and the refinement leads to significantly poorer reliability factors.

## Related literature

For related structures, see: Boeckmann & Näther (2010 ▶, 2011 ▶); Wriedt & Näther (2011 ▶); Wu *et al.* (2004 ▶). For a description of the Cambridge Structural Database, see: Allen (2002 ▶). 
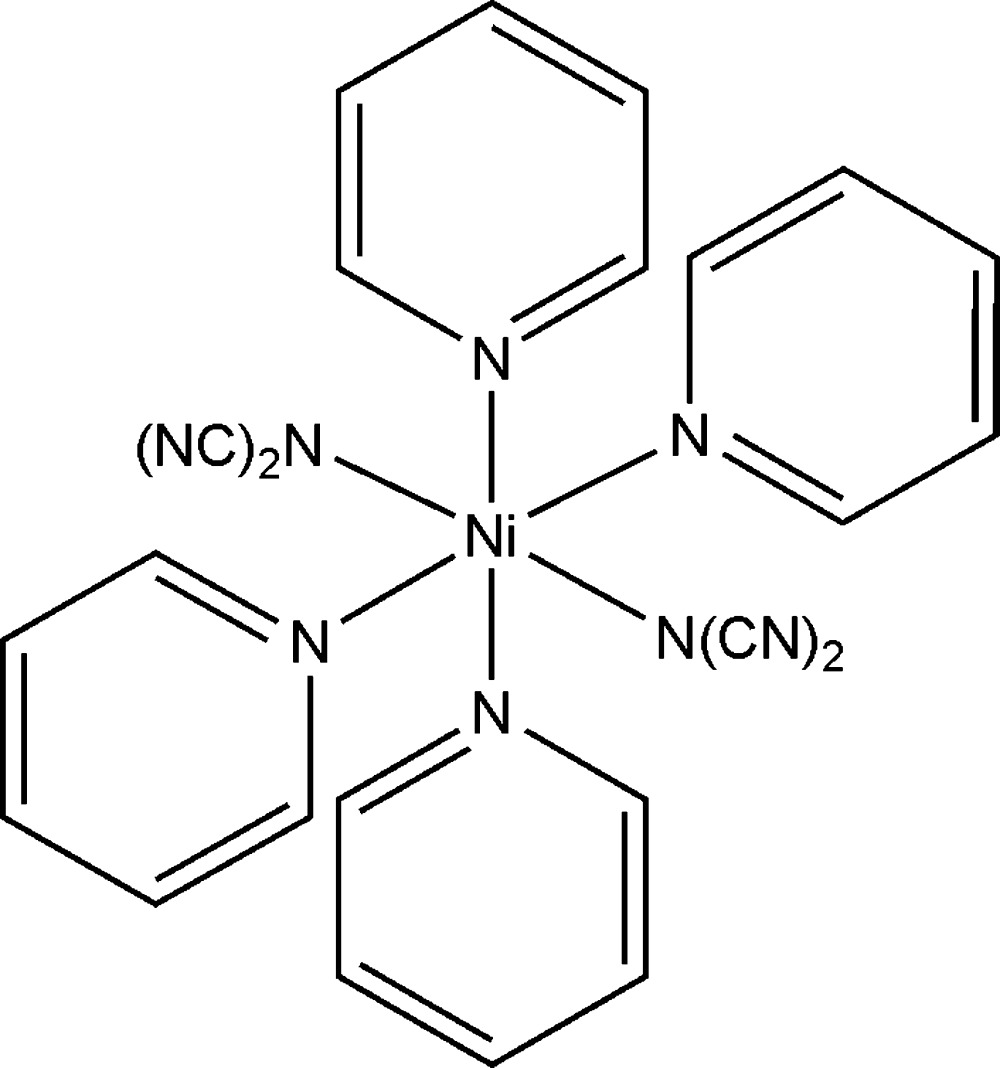


## Experimental

### 

#### Crystal data

[Ni(C_2_N_3_)_2_(C_5_H_5_N)_4_]
*M* *_r_* = 507.21
Monoclinic, 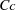

*a* = 13.0439 (6) Å
*b* = 12.8557 (8) Å
*c* = 15.1294 (7) Å
β = 110.191 (5)°
*V* = 2381.1 (2) Å^3^
*Z* = 4
Mo *K*α radiation
μ = 0.85 mm^−1^
*T* = 170 K
0.05 × 0.04 × 0.04 mm

#### Data collection

Stoe IPDS-1 diffractometer
Absorption correction: numerical (*X-SHAPE* and *X-RED32*; Stoe & Cie, 2008 ▶) *T*_min_ = 0.911, *T*_max_ = 0.973
11154 measured reflections
5386 independent reflections
4554 reflections with *I* > 2σ(*I*)
*R*_int_ = 0.035

#### Refinement

*R*[*F* ^2^ > 2σ(*F* ^2^)] = 0.036
*wR*(*F* ^2^) = 0.092
*S* = 0.98
5386 reflections
318 parameters
2 restraints
H-atom parameters constrained
Δρ_max_ = 0.35 e Å^−3^
Δρ_min_ = −0.44 e Å^−3^

### 

Data collection: *X-AREA* (Stoe & Cie, 2008 ▶); cell refinement: *X-AREA*; data reduction: *X-AREA*; program(s) used to solve structure: *SHELXS97* (Sheldrick, 2008 ▶); program(s) used to refine structure: *SHELXL97* (Sheldrick, 2008 ▶); molecular graphics: *XP* in *SHELXTL* (Sheldrick, 2008 ▶) and *DIAMOND* (Brandenburg, 2011 ▶); software used to prepare material for publication: *XCIF* in *SHELXTL*.

## Supplementary Material

Crystal structure: contains datablock(s) I, global. DOI: 10.1107/S1600536812019691/bt5887sup1.cif

Structure factors: contains datablock(s) I. DOI: 10.1107/S1600536812019691/bt5887Isup2.hkl

Additional supplementary materials:  crystallographic information; 3D view; checkCIF report

## Figures and Tables

**Fig. 1.**
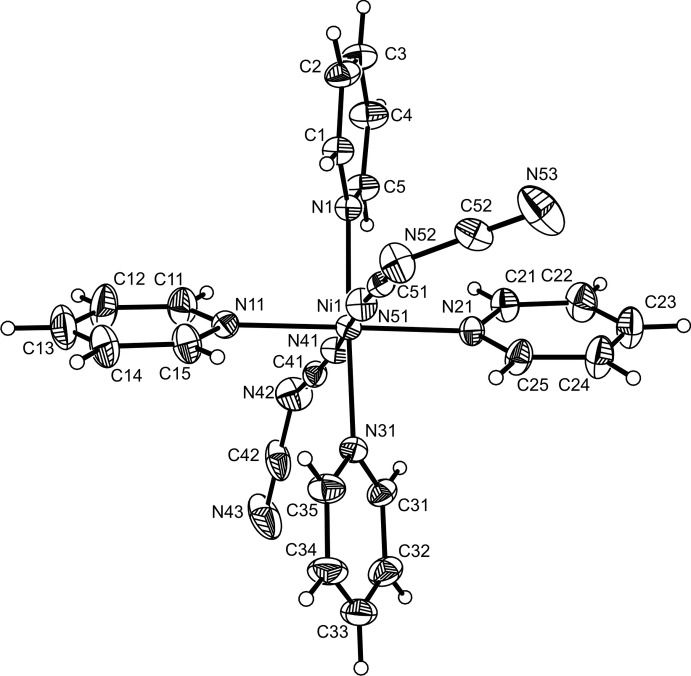
The molecular structure of the title compound with labelling and displacement ellipsoids drawn at the 50% probability level.

**Fig. 2.**
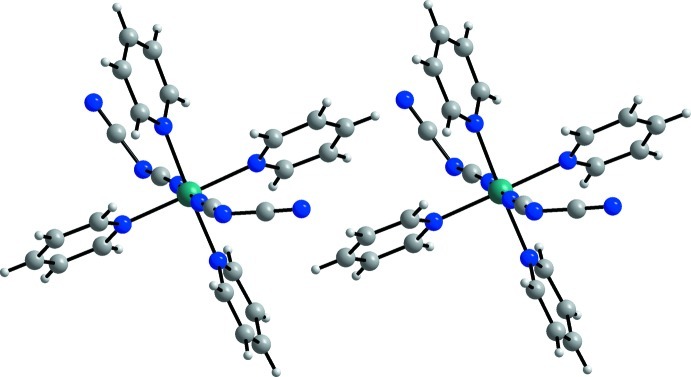
Crystal structure of the title compound with a view of the discrete complexes (green = nickel(II), blue = nitrogen, grey = carbon and white = hydrogen).

## supplementary crystallographic information

### Comment

Recently we have reported on the thermal and magnetic properties of coordination polymers based on paramagnetic transition metal thio- and selenocyanato compounds in which the cations are linked by thio- or selenocyanato anions (Boeckmann & Näther; 2010; Boeckmann & Näther, 2011). The bridging compounds with *e.g.* pyridine can be prepared by thermal decomposition of discrete complexes with terminal bonded anionic ligands. In the course of systematic investigations we also started to investigated similar compounds based on dicyanamide (Wriedt & Näther, 2011). Within this project crystals of the title compound were obtained, which were characterized by single-crystal X-ray diffraction. In the crystal structure of the title compound each nickel(II) cation is six-coordinated by two dicyanamido anions and four pyridine ligands in a slightly distorted octehedral geometry (Fig. 1). The NiN_6_ distances ranges from 2.057 (3) Å to 2.169 (3) Å and the angles are between 86.59 (11) ° and 178.96 (15)°. In the crystal structure, the discrete complexes are connected through intermolecular N—HC hydrogen bonds with an N···H distances of 2.559 Å (Fig. 2). The shortest nickel(II)—nickel(II) distance between the complexes is 9.157 Å.

It must be noted that a discrete nickel(II) dicyanamide complex is reported (Wu *et al.*, 2004) with 1,10-phenanthroline as co-ligands in which all ligands are *cis*-coordinated.

### Experimental

Nickel(II) chloride hexahydrate (NiCl_2_x6H_2_O), sodium dicyanamide (NaN(CN)_2_) and pyridine were obtained from Alfa Aesar. All chemicals were used without further purification. 0.125 mmol (29.7 mg) NiCl_2_x6H_2_O, 0.25 mmol (22.3 mg) Na(N(CN)_2_) were reacted in 2.5 ml pyridine. Light-green single-crystal of the title compound were obtained after three days.

### Refinement

All H atoms were located in difference map but were positioned with idealized geometry and were refined isotropic with *U*_eq_(H) = 1.2 *U*_eq_(C) of the parent atom using a riding model with C—H = 0.95 Å. The structure is pseudo-centrosymmetric and can also be refined in the centrosymmetric space group *C*2/*c*. However, in *C*2/*c* the complexes are located on centres of inversion and the anionic ligands are strongly disorderd which is not the case in space group *Cc*. Moreover, in *C*2/*c* the reliability factors are very poor and no reasonable structure model can be found. Therefore, *Cc* was selected, in which the structure can be very easily refined. In this case the absolute structure cannot be determined presumable, because of the pseudo-symmetry and therefore, a twin refinement for racemic twinning was performed leading to an BASF parameter of 0.53 (2).

### Crystal data

**Table:**

[Ni(C_2_N_3_)_2_(C_5_H_5_N)_4_]	*F*(000) = 1048
*M**_r_* = 507.21	*D*_x_ = 1.415 Mg m^−^^3^
Monoclinic, *C**c*	Mo *K*α radiation, λ = 0.71073 Å
Hall symbol: C -2yc	Cell parameters from 11154 reflections
*a* = 13.0439 (6) Å	θ = 3.0–28.1°
*b* = 12.8557 (8) Å	µ = 0.85 mm^−^^1^
*c* = 15.1294 (7) Å	*T* = 170 K
β = 110.191 (5)°	Block, light green
*V* = 2381.1 (2) Å^3^	0.05 × 0.04 × 0.04 mm
*Z* = 4	

### Data collection

**Table:**

Stoe IPDS-1 diffractometer	5386 independent reflections
Radiation source: fine-focus sealed tube	4554 reflections with *I* > 2σ(*I*)
Graphite monochromator	*R*_int_ = 0.035
phi scan	θ_max_ = 28.1°, θ_min_ = 3.0°
Absorption correction: numerical (*X-SHAPE* and *X-RED32*; Stoe & Cie, 2008)	*h* = −17→17
*T*_min_ = 0.911, *T*_max_ = 0.973	*k* = −16→16
11154 measured reflections	*l* = −19→19

### Refinement

**Table:**

Refinement on *F*^2^	Secondary atom site location: difference Fourier map
Least-squares matrix: full	Hydrogen site location: inferred from neighbouring sites
*R*[*F*^2^ > 2σ(*F*^2^)] = 0.036	H-atom parameters constrained
*wR*(*F*^2^) = 0.092	*w* = 1/[σ^2^(*F*_o_^2^) + (0.0593*P*)^2^] where *P* = (*F*_o_^2^ + 2*F*_c_^2^)/3
*S* = 0.98	(Δ/σ)_max_ < 0.001
5386 reflections	Δρ_max_ = 0.35 e Å^−^^3^
318 parameters	Δρ_min_ = −0.44 e Å^−^^3^
2 restraints	Extinction correction: *SHELXL97* (Sheldrick, 2008), Fc^*^=kFc[1+0.001xFc^2^λ^3^/sin(2θ)]^-1/4^
Primary atom site location: structure-invariant direct methods	Extinction coefficient: 0.0106 (6)

### Special details

**Table:**

Geometry. All e.s.d.'s (except the e.s.d. in the dihedral angle between two l.s. planes) are estimated using the full covariance matrix. The cell e.s.d.'s are taken into account individually in the estimation of e.s.d.'s in distances, angles and torsion angles; correlations between e.s.d.'s in cell parameters are only used when they are defined by crystal symmetry. An approximate (isotropic) treatment of cell e.s.d.'s is used for estimating e.s.d.'s involving l.s. planes.
Refinement. Refinement of *F*^2^ against ALL reflections. The weighted *R*-factor *wR* and goodness of fit *S* are based on *F*^2^, conventional *R*-factors *R* are based on *F*, with *F* set to zero for negative *F*^2^. The threshold expression of *F*^2^ > σ(*F*^2^) is used only for calculating *R*-factors(gt) *etc*. and is not relevant to the choice of reflections for refinement. *R*-factors based on *F*^2^ are statistically about twice as large as those based on *F*, and *R*- factors based on ALL data will be even larger.

### Fractional atomic coordinates and isotropic or equivalent isotropic displacement parameters (Å^2^)

**Table:**

	*x*	*y*	*z*	*U*_iso_*/*U*_eq_	
Ni1	0.53427 (5)	0.75512 (3)	0.65840 (5)	0.01803 (10)	
N1	0.5772 (2)	0.68096 (18)	0.7947 (2)	0.0204 (6)	
C1	0.5414 (3)	0.7163 (3)	0.8622 (3)	0.0254 (7)	
H1	0.4997	0.7786	0.8508	0.031*	
C2	0.5620 (3)	0.6669 (3)	0.9474 (3)	0.0314 (8)	
H2	0.5360	0.6956	0.9935	0.038*	
C3	0.6207 (3)	0.5749 (3)	0.9651 (3)	0.0332 (8)	
H3	0.6345	0.5385	1.0227	0.040*	
C4	0.6587 (3)	0.5376 (3)	0.8962 (3)	0.0338 (8)	
H4	0.6992	0.4747	0.9057	0.041*	
C5	0.6369 (3)	0.5932 (2)	0.8131 (3)	0.0280 (7)	
H5	0.6655	0.5682	0.7672	0.034*	
N11	0.6453 (2)	0.88091 (19)	0.7206 (2)	0.0230 (6)	
C11	0.7525 (3)	0.8647 (3)	0.7619 (3)	0.0364 (9)	
H11	0.7792	0.7954	0.7664	0.044*	
C12	0.8266 (4)	0.9446 (3)	0.7983 (4)	0.0512 (12)	
H12	0.9024	0.9299	0.8262	0.061*	
C13	0.7897 (4)	1.0450 (3)	0.7938 (4)	0.0480 (11)	
H13	0.8391	1.1008	0.8188	0.058*	
C14	0.6809 (4)	1.0628 (3)	0.7529 (3)	0.0416 (10)	
H14	0.6527	1.1315	0.7484	0.050*	
C15	0.6115 (3)	0.9793 (2)	0.7179 (3)	0.0331 (8)	
H15	0.5355	0.9929	0.6903	0.040*	
N21	0.4158 (2)	0.63412 (19)	0.5948 (2)	0.0223 (6)	
C21	0.4425 (3)	0.5333 (2)	0.6051 (3)	0.0285 (8)	
H21	0.5166	0.5153	0.6378	0.034*	
C22	0.3677 (4)	0.4536 (3)	0.5706 (3)	0.0403 (10)	
H22	0.3902	0.3829	0.5799	0.048*	
C23	0.2604 (4)	0.4788 (3)	0.5226 (3)	0.0419 (10)	
H23	0.2071	0.4259	0.4988	0.050*	
C24	0.2317 (3)	0.5822 (3)	0.5098 (3)	0.0406 (9)	
H24	0.1585	0.6020	0.4756	0.049*	
C25	0.3110 (3)	0.6567 (3)	0.5475 (3)	0.0297 (8)	
H25	0.2901	0.7279	0.5393	0.036*	
N31	0.4925 (2)	0.81948 (19)	0.5191 (2)	0.0228 (6)	
C31	0.5106 (3)	0.7632 (3)	0.4508 (3)	0.0269 (7)	
H31	0.5396	0.6950	0.4657	0.032*	
C32	0.4890 (3)	0.7998 (3)	0.3597 (3)	0.0359 (8)	
H32	0.5032	0.7575	0.3138	0.043*	
C33	0.4467 (3)	0.8983 (3)	0.3369 (3)	0.0368 (9)	
H33	0.4307	0.9249	0.2750	0.044*	
C34	0.4282 (4)	0.9574 (3)	0.4057 (3)	0.0384 (9)	
H34	0.4000	1.0260	0.3924	0.046*	
C35	0.4513 (3)	0.9153 (3)	0.4948 (3)	0.0326 (8)	
H35	0.4371	0.9565	0.5414	0.039*	
N41	0.6587 (2)	0.67389 (19)	0.6335 (2)	0.0241 (6)	
C41	0.7342 (3)	0.6558 (2)	0.6128 (2)	0.0234 (7)	
N42	0.8215 (3)	0.6277 (3)	0.5954 (3)	0.0398 (8)	
C42	0.8367 (3)	0.6685 (3)	0.5206 (3)	0.0445 (10)	
N43	0.8561 (4)	0.6974 (3)	0.4559 (3)	0.0723 (14)	
N51	0.4106 (2)	0.8376 (2)	0.6812 (2)	0.0248 (6)	
C51	0.3343 (3)	0.8681 (2)	0.6955 (2)	0.0198 (6)	
N52	0.2530 (3)	0.9113 (2)	0.7117 (2)	0.0304 (6)	
C52	0.1669 (3)	0.8567 (2)	0.7082 (2)	0.0266 (6)	
N53	0.0854 (3)	0.8178 (3)	0.7044 (3)	0.0512 (9)	

### Atomic displacement parameters (Å^2^)

**Table:**

	*U*^11^	*U*^22^	*U*^33^	*U*^12^	*U*^13^	*U*^23^
Ni1	0.01894 (14)	0.01636 (15)	0.02019 (15)	0.00311 (14)	0.00853 (10)	−0.00008 (15)
N1	0.0215 (15)	0.0189 (11)	0.0215 (17)	0.0028 (9)	0.0083 (13)	0.0001 (10)
C1	0.0266 (16)	0.0255 (15)	0.0252 (19)	0.0042 (13)	0.0103 (15)	0.0020 (14)
C2	0.0326 (18)	0.0417 (18)	0.0224 (18)	0.0036 (14)	0.0127 (15)	−0.0012 (14)
C3	0.037 (2)	0.0382 (18)	0.024 (2)	0.0060 (14)	0.0090 (17)	0.0072 (14)
C4	0.043 (2)	0.0283 (15)	0.030 (2)	0.0167 (14)	0.0136 (17)	0.0102 (13)
C5	0.0342 (18)	0.0260 (14)	0.0257 (18)	0.0106 (12)	0.0127 (15)	0.0035 (12)
N11	0.0242 (15)	0.0221 (12)	0.0234 (16)	0.0002 (10)	0.0093 (13)	−0.0014 (10)
C11	0.033 (2)	0.0281 (16)	0.044 (3)	−0.0006 (13)	0.0074 (19)	−0.0027 (14)
C12	0.033 (2)	0.044 (2)	0.061 (3)	−0.0111 (17)	−0.003 (2)	−0.008 (2)
C13	0.051 (3)	0.0321 (18)	0.060 (3)	−0.0195 (17)	0.019 (2)	−0.0114 (18)
C14	0.048 (2)	0.0229 (15)	0.055 (3)	−0.0078 (15)	0.018 (2)	−0.0056 (15)
C15	0.035 (2)	0.0189 (14)	0.047 (2)	−0.0006 (13)	0.0160 (18)	−0.0047 (14)
N21	0.0225 (15)	0.0185 (11)	0.0259 (16)	−0.0004 (9)	0.0084 (13)	−0.0028 (10)
C21	0.0280 (17)	0.0188 (13)	0.038 (2)	0.0029 (12)	0.0106 (16)	−0.0011 (12)
C22	0.048 (2)	0.0187 (15)	0.052 (3)	−0.0014 (14)	0.014 (2)	−0.0050 (14)
C23	0.035 (2)	0.0349 (18)	0.050 (3)	−0.0111 (15)	0.0068 (19)	−0.0073 (16)
C24	0.0274 (19)	0.0347 (18)	0.050 (3)	−0.0043 (14)	0.0009 (18)	−0.0037 (16)
C25	0.0222 (16)	0.0257 (14)	0.034 (2)	0.0013 (12)	0.0006 (15)	−0.0023 (13)
N31	0.0239 (15)	0.0220 (12)	0.0221 (17)	0.0011 (10)	0.0075 (13)	0.0024 (10)
C31	0.0275 (16)	0.0328 (17)	0.0208 (18)	0.0002 (13)	0.0089 (14)	−0.0039 (13)
C32	0.0334 (19)	0.053 (2)	0.023 (2)	−0.0027 (16)	0.0118 (16)	−0.0039 (15)
C33	0.033 (2)	0.051 (2)	0.026 (2)	−0.0024 (15)	0.0106 (17)	0.0107 (15)
C34	0.043 (2)	0.0395 (19)	0.030 (2)	0.0065 (15)	0.0077 (18)	0.0153 (15)
C35	0.040 (2)	0.0297 (15)	0.029 (2)	0.0076 (13)	0.0123 (17)	0.0056 (12)
N41	0.0250 (15)	0.0243 (12)	0.0258 (17)	0.0077 (10)	0.0122 (14)	0.0019 (10)
C41	0.0243 (16)	0.0230 (14)	0.0232 (18)	−0.0021 (11)	0.0085 (14)	−0.0037 (11)
N42	0.0305 (17)	0.0533 (19)	0.045 (2)	0.0087 (14)	0.0245 (15)	−0.0011 (14)
C42	0.048 (2)	0.0416 (18)	0.060 (3)	−0.0219 (16)	0.039 (2)	−0.0256 (17)
N43	0.103 (3)	0.067 (3)	0.082 (3)	−0.046 (2)	0.076 (3)	−0.033 (2)
N51	0.0223 (15)	0.0225 (12)	0.0307 (18)	0.0055 (10)	0.0106 (14)	−0.0014 (10)
C51	0.0244 (15)	0.0164 (12)	0.0168 (15)	0.0023 (10)	0.0048 (12)	−0.0002 (10)
N52	0.0295 (14)	0.0243 (13)	0.0441 (18)	0.0036 (11)	0.0213 (13)	−0.0051 (11)
C52	0.0266 (15)	0.0241 (13)	0.0324 (18)	0.0097 (11)	0.0144 (13)	0.0082 (11)
N53	0.0377 (17)	0.0438 (17)	0.079 (3)	0.0064 (14)	0.0294 (18)	0.0222 (17)

### Geometric parameters (Å, º)

**Table:**

Ni1—N51	2.057 (3)	N21—C25	1.338 (5)
Ni1—N41	2.071 (3)	C21—C22	1.386 (5)
Ni1—N31	2.152 (3)	C21—H21	0.9500
Ni1—N11	2.158 (3)	C22—C23	1.375 (6)
Ni1—N1	2.162 (3)	C22—H22	0.9500
Ni1—N21	2.169 (3)	C23—C24	1.377 (6)
N1—C1	1.341 (4)	C23—H23	0.9500
N1—C5	1.344 (4)	C24—C25	1.381 (5)
C1—C2	1.377 (5)	C24—H24	0.9500
C1—H1	0.9500	C25—H25	0.9500
C2—C3	1.383 (5)	N31—C35	1.343 (4)
C2—H2	0.9500	N31—C31	1.348 (5)
C3—C4	1.385 (5)	C31—C32	1.391 (5)
C3—H3	0.9500	C31—H31	0.9500
C4—C5	1.388 (5)	C32—C33	1.376 (6)
C4—H4	0.9500	C32—H32	0.9500
C5—H5	0.9500	C33—C34	1.376 (6)
N11—C15	1.336 (4)	C33—H33	0.9500
N11—C11	1.337 (5)	C34—C35	1.385 (5)
C11—C12	1.388 (6)	C34—H34	0.9500
C11—H11	0.9500	C35—H35	0.9500
C12—C13	1.371 (6)	N41—C41	1.154 (4)
C12—H12	0.9500	C41—N42	1.306 (4)
C13—C14	1.358 (7)	N42—C42	1.322 (5)
C13—H13	0.9500	C42—N43	1.154 (5)
C14—C15	1.386 (5)	N51—C51	1.157 (4)
C14—H14	0.9500	C51—N52	1.294 (4)
C15—H15	0.9500	N52—C52	1.309 (4)
N21—C21	1.337 (4)	C52—N53	1.159 (4)
			
N51—Ni1—N41	178.96 (15)	N11—C15—C14	123.8 (4)
N51—Ni1—N31	91.03 (12)	N11—C15—H15	118.1
N41—Ni1—N31	88.00 (11)	C14—C15—H15	118.1
N51—Ni1—N11	89.29 (11)	C21—N21—C25	116.7 (3)
N41—Ni1—N11	90.40 (11)	C21—N21—Ni1	121.8 (3)
N31—Ni1—N11	92.51 (11)	C25—N21—Ni1	121.4 (2)
N51—Ni1—N1	91.56 (11)	N21—C21—C22	123.5 (4)
N41—Ni1—N1	89.43 (11)	N21—C21—H21	118.3
N31—Ni1—N1	176.39 (11)	C22—C21—H21	118.3
N11—Ni1—N1	90.04 (11)	C23—C22—C21	118.7 (3)
N51—Ni1—N21	87.97 (11)	C23—C22—H22	120.6
N41—Ni1—N21	92.33 (11)	C21—C22—H22	120.6
N31—Ni1—N21	86.59 (11)	C22—C23—C24	118.6 (4)
N11—Ni1—N21	177.09 (12)	C22—C23—H23	120.7
N1—Ni1—N21	90.98 (11)	C24—C23—H23	120.7
C1—N1—C5	117.1 (3)	C23—C24—C25	118.9 (4)
C1—N1—Ni1	122.3 (2)	C23—C24—H24	120.5
C5—N1—Ni1	120.5 (2)	C25—C24—H24	120.5
N1—C1—C2	123.3 (3)	N21—C25—C24	123.5 (3)
N1—C1—H1	118.3	N21—C25—H25	118.2
C2—C1—H1	118.3	C24—C25—H25	118.2
C1—C2—C3	119.5 (3)	C35—N31—C31	116.2 (3)
C1—C2—H2	120.3	C35—N31—Ni1	124.3 (2)
C3—C2—H2	120.3	C31—N31—Ni1	119.5 (2)
C2—C3—C4	117.9 (3)	N31—C31—C32	123.3 (4)
C2—C3—H3	121.1	N31—C31—H31	118.3
C4—C3—H3	121.1	C32—C31—H31	118.3
C3—C4—C5	119.3 (3)	C33—C32—C31	119.1 (4)
C3—C4—H4	120.3	C33—C32—H32	120.5
C5—C4—H4	120.3	C31—C32—H32	120.5
N1—C5—C4	122.8 (3)	C32—C33—C34	118.6 (4)
N1—C5—H5	118.6	C32—C33—H33	120.7
C4—C5—H5	118.6	C34—C33—H33	120.7
C15—N11—C11	116.3 (3)	C33—C34—C35	118.9 (4)
C15—N11—Ni1	122.1 (3)	C33—C34—H34	120.5
C11—N11—Ni1	121.6 (2)	C35—C34—H34	120.5
N11—C11—C12	122.9 (4)	N31—C35—C34	123.9 (4)
N11—C11—H11	118.5	N31—C35—H35	118.1
C12—C11—H11	118.5	C34—C35—H35	118.1
C13—C12—C11	119.5 (5)	C41—N41—Ni1	161.0 (3)
C13—C12—H12	120.2	N41—C41—N42	174.3 (4)
C11—C12—H12	120.2	C41—N42—C42	117.5 (4)
C14—C13—C12	118.4 (4)	N43—C42—N42	174.2 (5)
C14—C13—H13	120.8	C51—N51—Ni1	168.7 (3)
C12—C13—H13	120.8	N51—C51—N52	174.3 (3)
C13—C14—C15	119.1 (4)	C51—N52—C52	120.7 (3)
C13—C14—H14	120.5	N53—C52—N52	173.2 (3)
C15—C14—H14	120.5		
